# Replacement of Palm Oil with Soybean Acid Oil in Broiler Chicken Diet: Fat Digestibility and Lipid Class Content along the Intestinal Tract

**DOI:** 10.3390/ani11092586

**Published:** 2021-09-03

**Authors:** Beatriz Jimenez-Moya, Ana C. Barroeta, Francesc Guardiola, María Dolores Soler, Raquel Rodriguez-Sanchez, Roser Sala

**Affiliations:** 1 Animal Nutrition and Welfare Service (SNiBA), Animal and Food Science Department, Faculty of Veterinary, Universitat Autònoma de Barcelona, Edifici V, Travessera dels Turons, 08193 Bellaterra, Spain; Beatriz.Jimenez@uab.cat (B.J.-M.); Ana.Barroeta@uab.cat (A.C.B.); 2 Libifood Research Group, Departament de Nutrició, Ciències de l’Alimentació i Gastronomia, INSA-UB, XIA, Campus de l’Alimentació Torribera, Facultat de Farmàcia i Ciències de l’Alimentació, Universitat de Barcelona, Av Prat de la Riba, 171, 08921 Santa Coloma de Gramenet, Spain; fguardiola@ub.edu; 3 AviFeed Science, Department of Animal Production and Health Public Veterinary Health and Food Science and Technology, Facultad de Veterinaria, Universidad Cardenal Herrera-CEU, CEU Universities, Calle Tirant lo Blanch 7, 46115 Alfara del Patriarca, Spain; mariola@uchceu.es; 4 AgResearch Ltd., Ruakura Research Centre, 10 Bisley Road, Private Bag 3123, Hamilton 3214, New Zealand; rodriguezsan.raquel@gmail.com

**Keywords:** fat digestibility, lipid classes, free fatty acids, fat by-products, acid oils, broiler chickens, poultry, intestinal tract

## Abstract

**Simple Summary:**

Soybean acid oil is a by-product rich in free fatty acids obtained from the soybean oil refining industry. Its inclusion in chicken diet is a way for it to be upcycled and to reduce the cost of feed. Its high degree of unsaturation could enhance the absorption of saturated fatty acids from palm oil. The objective of this study was to assess the effect of replacing palm oil with increasing amounts of soybean acid oil on fat digestion and absorption in starter and grower chickens. The replacement of palm oil with soybean acid oil improved fat utilization in both 11 and 35-day-old broiler chickens. With the age, the contribution of the upper ileum to a better fat absorption increases. In grower chickens, soybean acid oil at 6% of total inclusion or a blend of palm oil with soybean acid oil (2:4, *w*/*w*) led to adequate fat utilization, similar to soybean oil at 6%. The results suggest that the use of soybean acid oil blended with palm oil is a good solution for inclusion in broiler chicken diet.

**Abstract:**

This study aimed to evaluate the replacement of palm oil (P) with increasing levels of soybean acid oil (SA), a by-product of soybean oil (S) refining, on lipid class content and fatty acid (FA) digestibility in the intestine and excreta of chickens at 11 and 35 days (d). Five experimental diets were obtained by supplementing a basal diet with 6% of P (P6), 6% of SA (SA6), 4% of P + 2% SA (P4-SA2), 2% of P + 4% of SA (P2-SA4) and 6% of S (S6). A total of 480 one-d-old female broiler chickens (Ross 308) were housed in metabolic cages (6 cages/treatment, with 16 birds/cage). Replacing P with SA improved fat absorption at 11 and 35 d (*p* < 0.05), but not feed AME values and saturated FA (SFA) digestibility at 11 d. As age increased, the absorption of SFA and free fatty acids (FFA) improved, and the contribution of the upper ileum to FA absorption increased (*p* < 0.05). At 35 d, SA6 (56% FFA) and P2-SA4 (40% FFA, 2.6 unsaturated-to-saturated FA ratio) could replace S6 without impairing fat utilization. The replacement of P with SA represents a suitable strategy to use this by-product.

## 1. Introduction

The inclusion of fat sources in poultry diet is a common practice as these ingredients have the highest energy value and also supply essential fatty acids. Among them, vegetable sources are widely used, soybean oil (S) being the most extensively included in broiler diets. The high cost of S, which is expected to rise even more in the coming years [1], is the main reason to search for cheaper alternative lipid sources [2,3,4]. Moreover, the use of ingredients that are unsuitable for human consumption in animal nutrition would allow animal food producers to advance toward circular agroindustry. During the S refining process, different by-products are generated that could be attractive alternative lipid sources to feed broiler chickens [5,6,7]. One of these is soybean acid oil (SA), which is derived from the chemical refining of S. It has a similar fatty acid (FA) profile to S, with a high unsaturated-to-saturated FA ratio (UFA:SFA), but due to its origin it has a higher content of free fatty acids (FFA) (59–66%, determined by titration; and 45–61%, determined as lipid class) and MIU (moisture, insoluble impurities, unsaponifiable matter) (5.3–9.0%) [8].

It is well established that dietary fat utilization increases as age and UFA:SFA of the diet increase [9,10,11,12], but the different lipid class (triacylglycerols, TAG; diacylglycerols, DAG; monoacylglycerols, MAG, and FFA) content of fat sources is also important [13]. A fat source with a high level of FFA has been negatively associated to fat utilization [14]. However, subsequent studies have shown that blends of S + SA up to 15% FFA in starter chicks and up to 35% FFA in grower-finisher chickens have no negative repercussions on fat utilization [15,16]. On the other hand, a recent study in 35-day-old broiler chickens reported the positive effect of blending a saturated acid oil (palm fatty acid distillate) with conventional unsaturated oil (S), when the blend had less than 30% FFA and the UFA:SFA was higher than 2.6 [17]. Regarding these factors, several studies suggest that dietary fat absorption is more affected by its saturation degree than its FFA content [10,15,17,18]. In this context, we hypothesized that the replacement of palm oil (P) with SA could improve the utilization of the former, a potential strategy being to use this by-product in broiler chicken diet. However, this could depend on the age of the bird. To date, only Blanch et al. [19] have studied the addition of SA blended with P as a conventional saturated source (50:50, *w*/*w*) in one-year roosters, and reported a positive synergism between both fat sources. However, to our knowledge, the effect of blending SA with P to feed broiler chickens has yet to be addressed.

Therefore, the main objective was to study the effect of P replacement with increasing levels of SA on fat digestion and absorption processes in broiler chickens at 11 days (d) and at 35 d. This was evaluated by the studying the dynamics of lipid class content and FA digestibility in the intestinal tract and excreta. This study furthers knowledge about the relationship between UFA:SFA and FFA content, and consequently the use of this by-product in broiler chicken diet.

## 2. Materials and Methods

The study was performed at the animal experimental facilities of the Servei de Granges i Camps Experimentals (Universitat Autònoma de Barcelona; Bellaterra, Barcelona, Spain). The experimental protocol was reviewed and approved by the Animal Ethics Committee of the Universitat Autònoma de Barcelona (CEEAH; number code: 3938), in accordance with European Union guidelines for the care and use of animals in research (2010/63/EU).

### 2.1. Experimental Fats and Diets

Soybean oil (S) and soybean acid oil (SA) were obtained from Bunge (Wormerveer, North Holland). Palm oil (P) was sourced from Lipidos Santiga S.A. (Santa Perpetua de Mogoda, Barcelona, Spain). Oil samples were analyzed as described in Jimenez-Moya et al. [17], the chemical analyses of the experimental fats are presented in Table 1.

The manufacturing and the analyses of experimental diets are described in Jimenez-Moya et al. [17]. Briefly, a basal diet was formulated to both minimize basal fat content and meet or exceed FEDNA’s (Fundación Española para el Desarrollo de la Nutrición Animal) requirements [20] (Table 2). Two feeding periods (in mash form) were performed: starter (d 0–d 22) and grower-finisher (d 23–d 35). An indigestible marker, titanium dioxide (TiO_2_), was included (5 g/kg) in the basal diet.

The experimental diets were obtained after including a 6% of experimental fat sources or blends to the basal diet in a mixer: P6 (6% of P), P4-SA2 (4% of P and 2% of SA), P2-SA4 (2% of P and 4% of SA), and SA6 (SA at 6%). A positive control diet (S6) including a 6% of S was used. The nutritive value of the experimental diets is shown in Table 3.

### 2.2. Birds and Management

A total of 480 d-old female broiler chickens (Ross 308) with uniform body weight (BW, 39.11 g ± 2.57 g) and wing-banded, were randomly housed in metabolic cages (0.61 m × 0.50 m × 0.40 m) with 16 birds per cage, with a grid floor and excreta collection tray, located in an environmentally controlled room. Each cage was assigned to one of the five experimental diets (six replicates per treatment). Throughout the experimental period temperature, humidity, ventilation, and illumination were automatically controlled, according to Ross 308 lineage management handbook [21]. Briefly, the temperature was 30 °C on arrival, 28 ± 1 °C during the first week, and was gradually reduced 2 °C each week until reaching 20 °C at 5 weeks of age. The illumination program provided was 24 h of light at Day 1, 23:1 h (light:dark) from Days 2 to 10, and 18:6 h from Day 11 until the end of the study. Feed and water were offered ad libitum.

### 2.3. Controls and Sampling

At 11, 22 and 35 d of age individual BW and feed intake by cage were recorded, to calculate the average daily gain (ADG), average daily feed intake (ADFI) and the feed conversion ratio (FCR) of each period, and the entire experimental period results of the study. Mortality was recorded daily to adjust these parameters.

The coefficients of apparent digestibility of FA in each intestinal segment, as well as total tract digestibility (excreta) and energy, were analyzed twice, in the starter period from 9 to 11 d and in the grower-finisher period from 33 to 35 d using the same sampling procedure described in Jimenez-Moya et al. [17]. Briefly, samples of excreta were taken from each cage. Jejunum and ileum (12 birds per cage at 11 d and 2 birds per cage at 35 d) were excised and divided into 2 equidistant segments: upper and lower. The jejunum was considered as the portion of the small intestine from the end of the duodenum until the Meckel’s diverticulum, while the rest of the small intestine until 1 cm proximal to the ileocecal junction was considered the ileum segment. For each cage, the digestive contents from each individual segment were collected, pooled, and immediately frozen at −20 °C, until further lyophilization. Samples of diet, digesta and excreta were also kept at 4 °C until composition analyses. At the end of the experimental period (Day 35), the abdominal fat pad (from the proventriculus to the cloaca) was removed and weighed individually.

### 2.4. Laboratory Analyses and Calculations

Analytical determinations of the feed, intestinal content and excreta were performed as described in Jimenez-Moya et al. [17].

The digestibility coefficients of FA a were calculated by using the Ti ratio in the feed and digestive content or excreta. The apparent metabolizable energy (AME) was calculated by multiplying the apparent digestibility coefficient of the gross energy by its corresponding diet gross energy. The following formula [15] was used to estimate the content of each lipid class (TAG, DAG, MAG and FFA) present in the intestinal tract and excreta of the chickens:

Lipid class content = [LC]/[Ti],(1)

where [LC] is the concentration of each lipid class in the digesta of intestinal segment or excreta (mg/g DM) and [Ti] is the concentration of Ti in the digesta of intestinal segment or excreta (mg/g DM).

### 2.5. Statistical Analysis

The SAS statistical package (version 9.4, SAS Institute Inc., Cary, NC, USA) was used to analyze the data. Two main factors were considered: diet (5) × intestinal segment (5: upper and lower jejunum, upper and lower ileum, and excreta). Data were verified for normality and homogeneity of variance using the Shapiro–Wilk and Levene’s Tests, by using the UNIVARIATE procedure. All data were analyzed by one-way ANOVA using the General Linear Models (GLM) procedure, considering the cage as the experimental unit (*n* = 30; 5 treatments ×6 replicates). For each age, the effect of the diet on productive parameters and on feed AME values was tested. In addition, the effect of the diet, on lipid class content, FA digestibility, and on the contribution to FA absorption for each intestinal segment and excreta. At 35 d, the effect of the diet on abdominal fat deposition was also analyzed.

The effect of the intestinal segment on lipid class content was tested, with the intestinal segments and the excreta as the main factor (*n* = 150; 30 samples ×5 types of digesta samples), in both 11 and 35-d-old birds.

The effect of age (11 vs. 35 d) on the contribution of different intestinal segments to FA absorption was statistically analyzed using the age as the main factor (*n* = 60; 5 dietary treatments replicated 6 times ×2 ages). Furthermore, the effect of age on feed AME, and on lipid class content and FA digestibility at lower ileum was tested (*n* = 12; 6 replicates of lower ileum ×2 ages) for each treatment.

Tukey’s correction for multiple comparisons was used to test differences between treatment means. The results shown in the tables are reported as least-square means. Differences were declared significant at *p* < 0.05 for all statistical analyses.

## 3. Results

### 3.1. Characterization of Experimental Oils and Diets

The chemical analysis of the experimental fats is shown in Table 1. The highest value of MIU (moisture, impurities and unsaponifiable matter) content was obtained for SA (5.3%). Regarding FA composition, S and SA showed similar FA profiles with high content of linoleic and oleic acids, while the main FA in P were palmitic and oleic acids. The UFA:SFA was higher for S (5.29) and SA (4.02) than for P (0.98). Regarding lipid class content, S and P showed a higher TAG content (>92%), while SA was richer in FFA (61.2%).

As can be observed in Table 3, in both in starter and grower-finisher diets, the replacement of P with SA resulted in a progressive increase in the content of dietary FFA (from 9% to 56%), and consequently, a decrease in the TAG (from 79% to 28%). In parallel, an increase in UFA:SFA was also obtained, from 1.3 to 3.8.

### 3.2. Growth Performance and Abdominal Fat Deposition

Results from growth-performance and abdominal fat deposition in chickens fed various dietary treatments are presented in Table 4. The diet had no effect (*p* > 0.05) on any performance parameter in the starter period (from 0 to 22 d), or in the entire experimental period (from 0 to 35 d). However, in the grower-finisher period (from 23 to 35 d), chickens fed P6 or P4-SA2 had higher FCR than chickens fed S6 (*p* = 0.018).

Regarding the effect of the diet on fat deposition, differences were only obtained between chickens fed diets containing fat blends. Abdominal fat depot was higher in those chickens fed P4-SA2 than in birds fed P2-SA4 (*p* = 0.018).

### 3.3. Lipid Class Content in Different Intestinal Segments and Excreta

The lipid class content (TAG, DAG, MAG, FFA) in the intestinal tract and excreta in 11-d-old and 35-d-old broiler chickens are shown in Table 5 and Table 6, respectively. In both ages, FFA was the main lipid class present in the digesta and excreta. In 11-d-old chicks, a decrease in TAG, DAG, and FFA content was observed along the jejunum (*p* < 0.001) (Supplementary Table S1). In 35-d-old chickens, the content of all lipid classes decreased from the upper jejunum to upper ileum (*p* < 0.001) (Supplementary Table S1).

When P is replaced with graded levels of SA, higher TAG content was observed at 11 d (Table 5) in the lower jejunum (*p* = 0.004) and lower ileum (*p* = 0.028), and at 35 d (Table 6) in the upper and lower ileum and in the excreta (*p* ≤ 0.001). Similarly, the replacement of P with SA increased MAG content in starter chicks from the lower jejunum on (*p* < 0.001) and in grower chickens in the jejunum, upper ileum, and in the excreta (*p* ≤ 0.013).

Higher TAG content was observed in chickens fed SA6 compared to chickens fed S6 in the lower jejunum at 11 d (*p* = 0.004) and in the lower ileum and excreta at 35 d (*p* ≤ 0.001). The MAG content was also higher in chickens fed SA6 than those fed S6, the values of the latter being similar to those obtained in chickens fed P6; this was observed in starter chicks from the lower jejunum on (*p* < 0.001), and in grower chickens from the upper ileum on (*p* ≤ 0.029).

**Figure 1:**
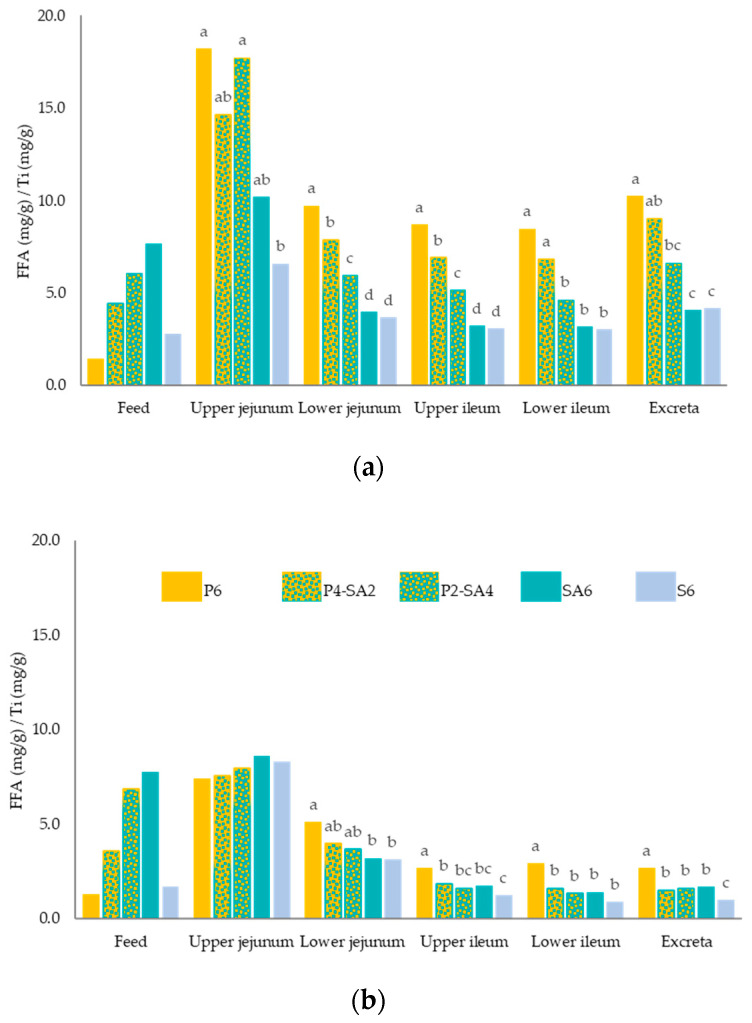
Free fatty acids (FFA) content^1^ in the feed, upper jejunum, lower jejunum, upper ileum, lower ileum, and excreta for the five different diets; with 6% of palm oil (P6), blend with 4% palm oil +2% soybean acid oil (P4-SA2), blend with 2% palm oil +4% soybean acid oil (P2-SA4), with 6% of soybean acid oil (SA6) and with 6% soybean oil (S6) in (**a**) 11-d-old broiler chickens and (**b**) 35-d-old broiler chickens. ^1^ FFA concentration (mg/g)/Ti concentration (mg/g) in each intestinal segment and excreta. Per each diet, means values are obtained from means of 6 replicates with 12 chickens/replicate at 11 d, and 2 chickens/replicate at 35 d. a–d: for each intestinal segment and excreta, different letter indicates significant differences (*p* ≤ 0.05).

Regarding FFA content in starter chicks (Table 5 and Figure 1a) and grower-finisher chickens (Table 6 and Figure 1b), lower FFA content was obtained as P was replaced with SA from the lower jejunum on at 11 d (*p* ≤ 0.001) and at 35 d (*p* ≤ 0.007). Those birds fed P6 diet showed the highest FFA content in the lower jejunum and upper ileum at 11 d, and from the upper ileum on at 35 d. In both starter and grower chickens, no differences were observed in the FFA content in any intestinal segment between chickens fed the two different soybean fat sources diets (SA6 and S6). In addition, in grower chickens, no differences were observed in any intestinal segment and excreta between chickens fed blend diets (P4-SA2 or P2-SA4) and those fed SA6.

In the lower ileum, starter chicks showed higher content of all lipid classes compared to grower-finisher chickens fed the same dietary treatment (*p* ≤ 0.021), except in MAG content for those birds fed P6, for which no differences were observed regarding the age of the animal (Supplementary Table S2).

### 3.4. Apparent Fatty-Acid Digestibility in Different Intestinal Segments and Excreta

The apparent FA digestibility coefficients determined in the different intestinal segments and in the excreta, and the feed apparent metabolizable energy for the different dietary treatments are shown in Table 7 and Table 8, for 11-d-old and 35-d-old broiler chickens, respectively.

Considering the effect of diet on the AME, differences were found between the different diets in both periods, from 0 to 21 d (*p* < 0.001) and from 22 to 35 d (*p* = 0.008). In the starter phase, no differences were observed among P6, P4-SA2, and P2-SA4. Regarding the unsaturated diets, S6 had higher AME than SA6. In the grower-finisher phase, P2-SA4 showed a higher AME value than SA6 and P6. The feed AME values of P6, blend diets, and SA6 were higher in the grower-finisher phase than in the starter phase (*p* ≤ 0.001) (Supplementary Table S3).

Regarding the apparent FA digestibility results, replacing P with SA increased the digestibility of TFA for both starter chicks (11 d) from the lower jejunum on (*p* < 0.001) and grower-finisher chickens from the upper ileum on (*p* ≤ 0.001). Similarly, the replacement of P with SA increased PUFA digestibility coefficients at 11 d in all intestinal segments (*p* ≤ 0.001) and at 35 d from the lower jejunum on (*p* < 0.001). In contrast, no effect on SFA digestibility coefficients was observed in starter chicks, whereas in grower chickens replacing P with SA increased the digestibility of SFA at the lower ileum level (*p* < 0.001). In the upper ileum, 35-d-old chickens fed P2-SA4 had the same SFA digestibility coefficients as those birds fed S6 and higher ones than those fed SA6 and P6 (*p* < 0.001).

Considering the results obtained at the lower ileum level, at 11 d chicks fed P2-SA4, SA6, and S6 did not differ for TFA, MUFA and PUFA, but for SFA those birds fed S6 showed the highest digestibility coefficients (*p* < 0.001). Grower chickens fed P4-SA2, P2-SA4, SA6, and S6 had no differences for TFA, SFA and MUFA values in the lower ileum, however for the digestibility of PUFA the lack of difference was observed among chickens fed P2-SA4, SA6, and S6.

Regarding the effect of age, the digestibility coefficients of TFA, SFA, and MUFA obtained in the lower ileum were higher in grower chickens than in starter chicks fed each dietary treatment (*p* < 0.001) (Supplementary Table S3). Similarly, higher digestibility of PUFA was observed at 35 d compared to 11 d in those animals fed S6 and blend diets (*p* ≤ 0.014) (Supplementary Table S3).

### 3.5. Contribution of Each Intestinal Segment to FA Digestibility

**Figure 2:**
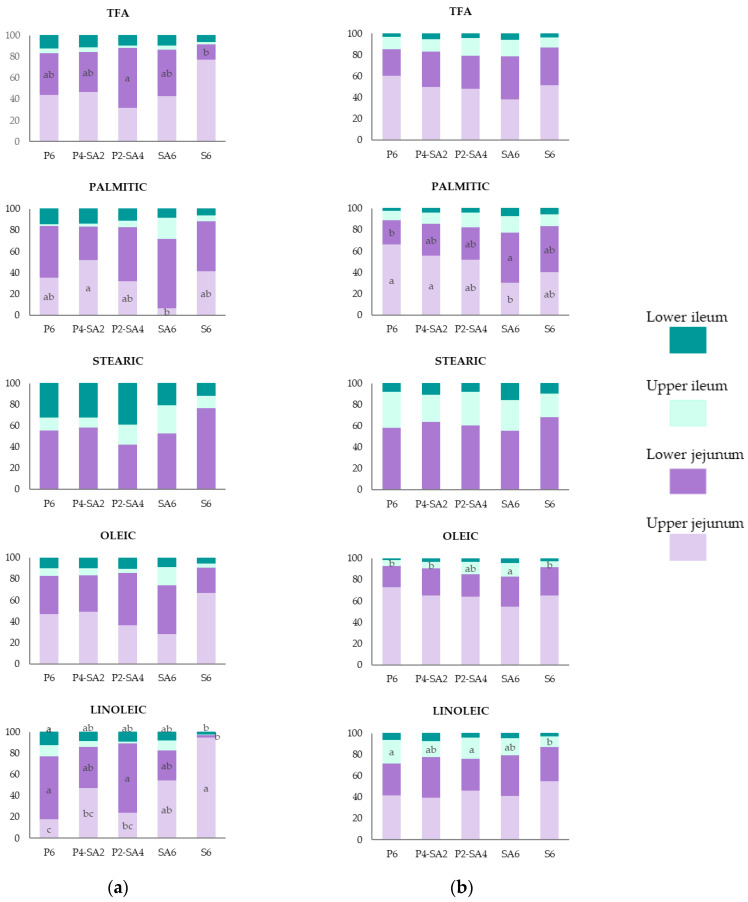
Contribution of each intestinal segment to the apparent fatty acid digestibility, calculated as a proportion of total digestibility reached at the lower ileum, along the intestinal tract for the five different diets; with 6% of palm oil (P6), blend with 4% palm oil +2% soybean acid oil (P4-SA2), blend with 2% palm oil +4% soybean acid oil (P2-SA4), with 6% of soybean acid oil (SA6) and with 6% soybean oil (S6) in (**a**) 11-d-old broiler chickens and (**b**) 35-d-old broiler chickens. TFA (total fatty acids), PALMITIC (C16:0), STEARIC (C18:0), OLEIC (C18:1 n-9) and LINOLEIC (C18:2 n-6) acids. Values are means of 6 replicates per each diet with 12 chickens/replicate at 11 d, and 2 chickens/replicate at 35 d. ^a–c^: within the same intestinal segment, columns not sharing a common letter are significantly different (*p* < 0.01).

Figure 2 shows the contribution of each intestinal segment to the digestibility of TFA, palmitic acid, stearic acid, oleic acid and linoleic acid, in relation to the digestibility reached in the lower ileum, for both 11-d- and 35-d-old broiler chickens.

Regardless of age, the jejunum was the intestinal segment where most TFA absorption took place (84%, on average of all diets at both ages). It was also similar for the absorption of palmitic acid (≥72% at 11 d; ≥78% at 35 d), oleic acid (≥74% at 11 d; ≥83% at 35 d), and linoleic acid (≥77% at 11 d; ≥72% at 35 d). No absorption of stearic acid was observed in the upper jejunum, but the contribution of the lower jejunum, on average for all diets, was 57% at 11 d, and 61% at 35 d.

The contribution of the different intestinal segments to the absorption of TFA for both starter and grower-finisher chickens was not affected by the replacement of P with SA, or by FFA content when SA6 was compared to S6; however, differences were observed for the absorption of individual FA. The results at 11 d (Figure 2a) show that the inclusion of SA as a replacement for P increased the absorption of linoleic acid at the upper jejunum level (*p* < 0.001). The absorption of linoleic acid in S6 occurred mainly in the upper jejunum, whereas this segment made a lower contribution in those diets that included P, blend diets and P6 (*p* < 0.001). In grower-finisher broiler chickens (Figure 2b) the replacement of P with SA delayed the absorption of palmitic acid, the absorption rate in SA6 compared to P6 was lower in the upper jejunum (*p* = 0.004) and inversely higher in the lower jejunum (*p* = 0.022). Similarly, the absorption of oleic acid in the upper ileum was higher when P was replaced with SA (*p* = 0.002).

In general, the contribution of the lower ileum to the absorption of TFA and individual FA was higher for starter chicks than for grower chickens (*p* ≤ 0.002) (Supplementary Table S4). The contribution of the upper ileum was greater at 35 d than at 11 d, mainly due to the absorption of stearic and linoleic acid (*p* ≤ 0.007) (Supplementary Table S4). Differences between ages were also observed in the jejunum; the absorption of palmitic acid and oleic acid was higher in the upper jejunum at 35 d (*p* ≤ 0.019), whereas the absorption of these FA was higher in the lower jejunum at 11 d (*p* ≤ 0.005) (Supplementary Table S4).

## 4. Discussion

The results obtained show that the lipolysis process in 11-day-old chickens took place until the lower jejunum, whereas in 35-day-old chickens it reached the upper ileum. The lower TAG content observed in the ileum at 35 d confirms the improvement in the capacity of the hydrolysis process depending on the age of the bird, in accordance with results obtained in our previous study [17]. This has been attributed to several factors: an insufficient emulsification process due to less mature gastrointestinal tract (GIT) in starter chicks compared to grower chickens [22], an increase in the rate of bile secretion with age [23] and a less efficient turnover of bile acids in young chicks than in older ones [24]. From the results obtained, no relation between TAG content in the feed and those obtained through the intestinal segments was observed. In this regard, Rodriguez-Sanchez et al. [15] determined the lipid classes in the duodenum, where the hydrolysis process mainly takes place [12], and reported that the dietary FFA% did not affect the hydrolysis process in 14-d-old chicks.

Evaluation of the absorption process is based on the results of the lipid classes, in particular the decrease in FFA content in the intestine together with the increase in apparent FA absorption. It is well known that no FA absorption occurs after the lower ileum [25] and the results obtained in the excreta could be affected by the FA production from the microbiota [26] as has been suggested by Rodriguez-Sanchez et al. [15]. Therefore, the results obtained from the lower ileum are considered to be the maximum FA digestibility coefficients.

The results showed that the replacement of P with SA, increasing the UFA:SFA as well as the FFA level of the diet, had a positive effect on dietary fat utilization, improving the digestibility of TFA and PUFA at both ages, and the digestibility of SFA at 35 d. The data also showed that young broilers were not fully capable of utilizing SA; the detriment was mainly obtained in SFA absorption, the digestibility coefficients of SA6 being similar to P6. However, as the age of the chicken increased, the utilization of SA6 improved; 35-d-old chickens fed diets containing SA reached higher SFA digestibility in the lower ileum than those birds fed P6 diet.

However, the comparison between chickens fed SA6 and those birds fed S6 gives us information about the effect of the dietary FFA level, as both diets had a similar UFA:SFA. The dietary FFA level only had a negative repercussion on the feed AME values and the SFA absorption in 11-d-old broiler chicks obtained in the lower ileum. This could be associated with the formation of insoluble calcium soaps, between SFA in free form and Ca^2+^, decreasing its availability to be absorbed and precipitating in the feces [27], as found in a previous in vitro study in which SA showed higher precipitated fat content than S [18]. Moreover, the poorly developed GIT in young chicks, mainly the limited bile salts content, and the lower utilization of MAG in SA6 compared to S6 (Table 5), which are both necessary to form dietary mixed micelles (DMM), could also explain the lower digestibility of SFA observed. The negative effect of dietary FFA disappeared with age, suggesting that the use of SA (56% FFA) in grower-finisher diets could be a suitable and economic alternative energy source, in accordance with Borsatti et al. [5]. Results for FFA absorption and FA digestibility in the lower ileum using SA6 were similar to those obtained with S6, as they also were for the performance parameters, abdominal fat deposition, and feed AME values. Therefore, the potential inclusion of this acid oil depends on the age of the bird, which agrees with Rodriguez-Sanchez et al. [15], Jimenez-Moya et al. [17], and Rodriguez-Sanchez et al. [16].

An interesting result was observed in P2-SA4 grower-finisher diet for the feed AME values; the feed AME values obtained were 107 kcal higher than those calculated from the value for the two separate sources in the blended proportion. That result obtained between P and SA agrees with the results reported by Blanch et al. [19], who added a blend of fat sources (2% P + 2% SA) to a basal diet in one-year-old roosters. The positive effect of blending a saturated oil with an unsaturated acid oil could be explained by the associative effect of blending a higher proportion of UFA with a lower proportion of SFA and the positive effect of increasing the ratio of TAG and DAG to FFA, compared to SA, as discussed in previous studies [9,13,17,28].

The results obtained using SA (56% FFA; 4 UFA:SFA) and the positive results obtained with the P2-SA4 blend (40% FFA; 2.6 UFA:SFA) in the grower-finisher broiler feeding phase suggest that it is possible to include a high FFA level in the diet if it comes from an unsaturated source. In our previous study [17], using a saturated acid oil (palm fatty acid distillate, PFAD) blended with S, a positive result was reported when the FFA content did not exceed 30% and the blend also had a 2.6 UFA:SFA. From the results of both studies, it could be suggested that at the same UFA:SFA chickens can better absorb FFA provided by unsaturated sources than saturated ones. This could be because unsaturated FFA from SA could easily be included in the DMM and thus become more bioaccessible [18], whereas saturated FFA from PFAD could not be absorbed readily because they form more calcium soaps than the UFA [27].

The positive effect of FA utilization in grower-finisher birds, especially on the absorption of SFA, has been reported by Batal and Parsons, [29]; Tancharoenrat et al. [9]; Roll et al. [13]; Rodriguez-Sanchez et al. [12]; and Viñado et al. [6]. This is evidenced by the higher feed AME values and FA digestibility coefficients, and the lower FFA content obtained in grower chickens compared to starter chicks. The results of the present study confirmed that the age of the chicken improved dietary FFA utilization. This has been related to the greater capacity for fat digestion, and also when fats are rich in FFA as has been reported using PFAD [17]. This could be associated with a longer feed passage time along the GIT in adult broilers compared to young birds [30]. According to the results, the upper ileum was the intestinal segment in which the increase in the contribution to FA absorption was higher, and this could partly explain the improvement in fat utilization in grower chickens as compared to starter ones, as has been suggested previously [17].

In agreement with Tancharoenrat et al. [31] and Rodriguez-Sanchez et al. [12] results obtained evidenced that the jejunum is the intestinal segment where most of TFA absorption takes place. Its contribution ranged from 77% to 92%, in agreement with Jimenez-Moya et al. [17]. Almost half of the TFA absorption occurred in the upper jejunum (on average for all diets: 48% at 11 d; 50% at 35 d), around one third was absorbed in the lower jejunum (37% at 11 d; 33% at 35 d), and the absorption of the residual FA took place in the upper and lower ileum, the magnitude of which was affected by the age of the bird; upper ileum (6% at 11 d; 13% at 35 d) and lower ileum (9% at 11 d; 4% at 35 d). It is also remarkable to note the delayed absorption of stearic acid, starting in the lower jejunum, as described in Jimenez-Moya et al. [17]. Although both palmitic acid and stearic acid are saturated FA, the greater molar volume of the latter probably causes the difference in behavior since it could affect the inclusion rate in the DMM [32].

The results showed that the replacement of P with SA delayed palmitic acid absorption in both starter and grower chicks. In contrast, the absorption of linoleic acid in 11-d-old chicks was faster as more SA was included in replacement of P. This suggests that the concentration of each FA in the diet could determine the preference for their inclusion in the DMM, the absorption of a major FA being faster than the absorption of a minor FA (palmitic acid, SA:16% vs. P6:37%; linoleic acid, SA:53% vs. P6:21%; Table 3). Palmitic acid is also less soluble in bile salt solutions than linoleic acid [32].

## 5. Conclusions

The current study evidenced that FA absorption determined in the lower ileum was essential to understand the utilization of diets differing in terms of their UFA:SFA and FFA content. Therefore, lower ileum content should be considered for sampling for future fat digestibility studies. The results demonstrated the strong effect of the age of the bird on the hydrolysis and absorption processes of fats. At 35 days, the better absorption of FA by broiler chickens was mainly related to an improvement of both the utilization of FFA and mainly the dietary SFA, being the upper ileum the segment which the increase in the contribution to FA absorption was higher.

The blending of palm oil with soybean acid oil is a potential strategy for use of this by-product in both starter and grower-finisher diets, improving the absorption of palm oil, and obtaining similar fat utilization in grower-finisher chickens to the use of soybean oil. In the starter phase, soybean acid oil improved palm oil digestibility, although it did not achieve the fat digestibility obtained with the use of soybean oil. In the grower-finisher phase, a positive effect was obtained on AME blending 2% palm oil + 4% soybean acid oil. Soybean acid oil added at 6% to a basal diet or a blend of palm oil and soybean acid oil (2:4, *w*/*w*), with a dietary UFA:SFA of 2.6 and 40% FFA, could replace soybean oil in grower-finisher diets, without a negative repercussion on fat utilization.

The replacement of palm oil with soybean acid oil or the inclusion of soybean acid oil as a unique fat source to feed broiler chickens provides a potential strategy to use this by-product, improving the absorption of palm oil, and obtaining similar fat utilization in grower-finisher chickens to the use of soybean oil.

## Figures and Tables

**Table 1:** Chemical analyses of the experimental fats ^1^.

Item	P	SA	S
Moisture (g/100 g)	ND	1.43	ND
Insoluble impurities (g/100 g)	0.59	1.57	1.27
Unsaponifiable matter (g/100 g)	0.21	2.34	0.99
Fatty acid composition (g/100 g) ^2^			
C16:0	43.94	14.89	10.98
C18:0	4.64	3.46	3.47
C18:1 n-9	38.43	21.06	25.11
C18:2 n-6	9.70	51.71	51.70
C18:3 n-3	0.13	5.31	5.34
Minor fatty acids ^3^	3.15	3.58	3.40
SFA	50.64	19.88	15.86
*cis*-MUFA	39.44	23.06	27.06
*trans*-C18:1	0.08	0.04	0.04
PUFA	9.83	57.02	57.04
UFA:SFA	0.98	4.02	5.29
Lipid class composition (g/100 g) ^4^			
TAG	92.46	25.32	96.27
DAG	7.54	13.48	3.23
MAG	ND	ND	ND
FFA	ND	61.20	0.50
T (mg/kg)	199.40	1464.25	1007.31
T3 (mg/kg)	431.87	8.78	ND

Abbreviations: P, palm oil; SA, soybean acid oil; S, soybean oil; SFA, saturated fatty acids; MUFA, monounsaturated fatty acids; PUFA, polyunsaturated fatty acids; UFA:SFA, unsaturated to saturated fatty acid ratio, calculated as described by Varona et al. [8]; TAG, triacylglycerols; DAG, diacylglycerols; MAG, monoacylglycerols; FFA, free fatty acids; T, sum of α-, β-, γ- and δ-tocopherols; T3, sum of α-, β-, γ- and δ-tocotrienols; ND, not detected. ^1^ Chemical composition analyzed as described by Varona et al., [8]. ^2^ Percentage of total fatty acids (peak area normalization, %); ^3^ Minor fatty acids identified and quantified: C6:0, C8:0, C10:0, C11:0, C12:0, C13:0, C14:0, C15:0, C16:1 n-9, C16:1 n-7, C17:0, *trans*-C18:1, C18:1 n-7, C20:0, C20:1 n-9, C21:0, C20:2 n-6, C22:0, C23:0, C22:2, C24:0. ^4^ Percentage of total lipid classes (peak area normalization, %).

**Table 2:** Ingredient composition of the experimental basal diet [17].

Ingredients (g/kg)	Starter Diet (from 0 d to 22 d)	Grower-Finisher Diet (from 23 d to 35 d)
Wheat	544.9	440.2
Soybean meal 47%	354.0	272.5
Barley	-	185.8
Experimental fats ^1^	60.0	60.0
Calcium carbonate	14.4	13.9
Monocalcium phosphate	9.9	12.0
Titanium dioxide	5.0	5.0
Vitamin and mineral premix ^2^	4.0	4.0
Sodium chloride	4.0	3.5
DL-Methionine	2.3	1.7
L-Lysine	1.5	1.2
L-Threonine	-	0.2

^1^ Soybean oil, soybean acid oil and palm oil in different proportions. ^2^ Provides per kg of feed: vitamin A (from retinol), 10,000 IU; vitamin D3 (from cholecalciferol), 4800 IU; vitamin E (from alfa tocopherol), 45 mg; vitamin B1, 3 mg; vitamin B2, 9 mg; vitamin B6, 4.5 mg; vitamin B12, 40 µg; vitamin K3, 3 mg; calcium pantothenate, 16.5 mg; nicotinic acid, 51 mg; folic acid, 1.8 mg; biotin, 150 µg; Fe (from FeSO_4_·7H_2_O), 54 mg; I (from Ca(I_2_O_3_)_2_), 1.2 mg; Cu (from CuSO_4_·5H_2_O), 12 mg; Mn (from MnO), 90 mg; Zn (from ZnO), 66 mg; Se (from Na_2_SeO_3_), 0.18 mg; β-glucanase 150 U; xylanase 270 U.

**Table 3:** The analyzed ^1^ nutritive value, fatty acid profile, and lipid class of the experimental diets ^2^.

Item	Starter Diets (from 0 to 22 d)					Grower-Finisher Diets (from 23 to 35 d)				
	P6	P4-SA2	P2-SA4	SA6	S6	P6	P4-SA2	P2-SA4	SA6	S6
Dry matter (g/kg)	909.3	911.9	907.0	910.0	910.0	900.2	902.7	902.5	911.6	901.4
Crude protein (g/kg)	231.5	234.9	239.5	238.1	236.1	208.4	213.4	205.7	217.7	210.4
Ether extract (g/kg)	77.0	76.2	74.3	73.8	75.1	74.9	77.1	76.9	75.6	81.8
Crude fiber (g/kg)	32.0	32.7	30.6	28.4	32.9	34.1	30.0	36.4	36.2	30.8
Crude ash (g/kg)	70.9	68.4	66.9	69.1	55.4	57.5	59.0	62.9	66.1	62.1
Gross energy, kcal/kg	4332	4345	4301	4325	4367	4324	4353	4320	4365	4339
Fatty acid composition (g/100 g of fat)										
C14:0	0.87	0.65	0.41	-	-	0.85	0.63	0.38	-	0.06
C16:0	37.38	30.51	23.53	15.71	14.43	36.85	30.16	22.84	15.66	13.24
C18:0	4.29	4.00	3.70	3.37	3.48	4.17	3.91	3.62	3.33	3.35
C18:1 n-9	32.34	28.26	24.04	19.57	22.83	32.49	28.34	24.06	19.60	22.61
C18:1 n-7	0.83	1.06	1.30	1.57	1.46	0.80	1.05	1.30	1.55	1.50
C18:2 n-6	21.37	31.47	41.60	52.70	50.78	22.02	31.76	42.08	52.64	52.04
C18:3 n-3	1.46	2.70	3.98	5.35	5.27	1.60	2.82	4.12	5.42	5.50
Minor fatty acids ^3^	1.46	1.34	1.44	1.73	1.75	1.22	1.32	1.61	1.80	1.69
SFA	43.13	35.76	28.34	20.09	18.72	42.47	35.30	27.76	20.01	17.70
MUFA	34.04	30.06	26.08	21.87	25.24	33.92	30.12	26.05	21.93	24.76
PUFA	22.83	34.18	45.58	58.05	56.04	23.62	34.58	46.20	58.06	57.54
UFA:SFA	1.30	1.76	2.47	3.88	4.16	1.34	1.80	2.55	3.88	4.54
Lipid class composition (g/100 of fat)										
TAG	78.67	59.94	44.07	28.20	71.88	78.53	61.19	44.00	27.52	76.67
DAG	10.54	12.14	12.39	12.98	11.73	10.44	11.62	12.44	13.08	10.23
MAG	1.84	2.57	2.71	3.08	2.19	2.31	2.40	2.84	3.09	2.49
FFA	8.96	25.35	40.84	55.74	14.20	8.72	24.79	40.73	56.31	10.61

Abbreviations: SFA, saturated fatty acids; MUFA, monounsaturated fatty acids; PUFA, polyunsaturated fatty acids; UFA:SFA, unsaturated to saturated fatty acid ratio, calculated as described by Varona et al. [8]; TAG, triacylglycerols; DAG, diacylglycerols; MAG, monoacylglycerols; FFA, free fatty acids. ^1^ All samples were analyzed at least in duplicate. ^2^ Dietary treatments supplemented with 6% of palm oil (P), soybean acid oil (SA), soybean oil (S), or oil blends with 4% palm oil + 2% soybean acid oil (P4-SA2) or 2% palm oil + 4% soybean acid oil (P2-SA4). In all cases, fatty acids and lipid classes are expressed as peak area normalization (in %). ^3^ Minor fatty acids identified and quantified: C8:0, C10:0, C11:0, C12:0, C13:0, C14:1, C15:0, C15:1, C16:1, C17:0, C17:1, *trans*-C18:1, C18:3 n-6, C20:0, C20:1 n-9, C21:0, C20:2 n-6, C20:3 n-6, C20:4 n-6, C20:3 n-3, C20:5 n-3, C22:0, C22:1 n-9, C23:0, C22:2, C24:0, C22:6 n-3, C24:1.

**Table 4:** Growth performance and abdominal fat of broiler chickens fed various fat sources ^1^.

Dietary Treatments ^2^							
Item	P6	P4-SA2	P2-SA4	SA6	S6	SEM ^3^	*p*-Value
From 0 to 22 d							
ADFI, g/d/bird	54.5	52.3	52.2	51.0	48.7	2.43	0.554
ADG, g/d/bird	40.6	40.5	39.1	38.7	37.2	1.43	0.422
FCR, g/g	1.34	1.29	1.34	1.32	1.31	0.037	0.833
BW at 22 d, g	933	930	898	890	856	31.4	0.416
From 23 to 35 d							
ADFI, g/d/bird	143	145	142	139	134	2.90	0.077
ADG, g/d/bird	89.9	90.3	90.9	88.3	87.8	1.96	0.768
FCR, g/g	1.60 ^a^	1.61 ^a^	1.57 ^ab^	1.58 ^ab^	1.53 ^b^	0.017	0.018
BW at 35 d, g	2101	2104	2079	2038	1997	45.6	0.425
From 0 to 35 d							
ADFI, g/d/bird	87.5	86.9	85.7	83.8	80.3	2.28	0.199
ADG, g/d/bird	58.9	59.0	58.3	57.1	56.0	1.30	0.426
FCR, g/g	1.49	1.47	1.47	1.47	1.43	0.018	0.383
Abdominal fat, g	32.36 ^ab^	36.49 ^a^	27.84 ^b^	29.50 ^ab^	29.62 ^ab^	2.051	0.018
Abdominal fat, %	1.53 ^ab^	1.73 ^a^	1.33 ^b^	1.43 ^ab^	1.46 ^ab^	0.091	0.019

Abbreviations: ADFI, average daily feed intake; ADG, average daily gain; FCR, feed conversion ratio; BW, body weight. ^1^ Diets supplemented with 6% of palm oil (P), soybean acid oil (SA), soybean oil (S), or oil blends with 4% palm oil + 2% soybean acid oil (P4-SA2) or 2% palm oil + 4% soybean acid oil (P2-SA4). ^2^ Values are pooled means of 6 replicates with 16 chickens/replicate from 0 to 11 d and 4 chickens/replicate from 11 to 35 d. In the case of BW, values are means of 24 chickens each treatment from 22 to 35 d. For abdominal fat, values are means of 2 chickens/replicate: 12 for each treatment at 35 d. ^3^ SEM, standard error of means of 6 observations per treatment (the experimental unit is the cage). ^a,b^: means in a row not sharing a common letter are significantly different (*p* < 0.05).

**Table 5:** Lipid class content ^1^ in the different intestinal segments and excreta in 11-d-old broiler chickens fed various experimental fat sources in the diet ^2^.

Item	Dietary Treatments					SEM ^3^	*p*-Value
	P6	P4-SA2	P2-SA4	SA6	S6		
Upper Jejunum							
TAG	0.49	0.52	0.46	0.85	0.53	0.136	0.261
DAG	1.99	1.98	2.72	2.60	1.30	0.445	0.193
MAG	0.15	0.31	0.24	0.36	0.18	0.052	0.052
FFA	18.20 ^a^	14.64 ^ab^	17.72 ^a^	10.18 ^ab^	6.58 ^b^	2.045	0.001
Lower Jejunum							
TAG	0.30 ^b^	0.42 ^ab^	0.45 ^ab^	0.54 ^a^	0.34 ^b^	0.041	0.004
DAG	0.76	0.92	0.98	0.98	0.82	0.092	0.285
MAG	0.17 ^c^	0.30 ^b^	0.42 ^ab^	0.46 ^a^	0.18 ^c^	0.029	<0.001
FFA	9.70 ^a^	7.89 ^b^	5.96 ^c^	3.96 ^d^	3.65 ^d^	0.415	<0.001
Upper Ileum							
TAG	0.24	0.30	0.32	0.34	0.25	0.050	0.522
DAG	0.62	0.58	0.73	0.69	0.64	0.099	0.843
MAG	0.13 ^d^	0.27 ^bc^	0.37 ^ab^	0.40 ^a^	0.16 ^cd^	0.026	<0.001
FFA	8.69 ^a^	6.90 ^b^	5.15 ^c^	3.22 ^d^	3.05 ^d^	0.414	<0.001
Lower Ileum							
TAG	0.19 ^b^	0.30 ^ab^	0.36 ^a^	0.38 ^a^	0.32 ^ab^	0.041	0.028
DAG	0.45	0.58	0.72	0.68	0.64	0.083	0.188
MAG	0.15 ^d^	0.35 ^bc^	0.45 ^ab^	0.52 ^a^	0.23 ^cd^	0.033	<0.001
FFA	8.47 ^a^	6.83 ^a^	4.63 ^b^	3.17 ^b^	3.02 ^b^	0.428	<0.001
Excreta							
TAG	0.29 ^b^	0.33 ^ab^	0.53 ^a^	0.37 ^ab^	0.38 ^ab^	0.044	0.034
DAG	0.80	1.08	1.07	0.88	0.98	0.167	0.515
MAG	0.21 ^b^	0.28 ^ab^	0.38 ^a^	0.43 ^a^	0.19 ^b^	0.040	<0.001
FFA	10.24 ^a^	9.01 ^ab^	6.62 ^bc^	4.05 ^c^	4.16 ^c^	0.631	<0.001

Abbreviations: TAG, triacylglycerols; DAG, diacylglycerols; MAG, monoacylglycerols; FFA, free fatty acids. ^1^ Lipid class concentration (mg/g)/Ti concentration (mg/g) in each intestinal segment and excreta. ^2^ Values are pooled means of 6 replicates with 12 chickens/replicate fed diets supplemented with 6% of palm oil (P), soybean acid oil (SA), soybean oil (S), or oil blends with 4% palm oil + 2% soybean acid oil (P4-SA2) or 2% palm oil + 4% soybean acid oil (P2-SA4). ^3^ SEM = standard error of the mean. ^a–d^: means in a row not sharing a common letter are significantly different (*p* < 0.05).

**Table 6:** Lipid class content ^1^ in the different intestinal segments and excreta in 35-d-old broiler chickens fed various experimental fat sources in the diet ^2^.

Item	Dietary Treatments					SEM ^3^	*p*-Value
	P6	P4-SA2	P2-SA4	SA6	S6		
Upper Jejunum							
TAG	0.26	0.25	0.19	0.34	0.21	0.053	0.255
DAG	1.20	1.38	1.63	1.83	1.32	0.169	0.097
MAG	0.17 ^b^	0.22 ^ab^	0.33 ^ab^	0.41 ^a^	0.24 ^ab^	0.047	0.011
FFA	7.40	7.56	7.99	8.59	8.28	0.816	0.830
Lower Jejunum							
TAG	0.22	0.21	0.27	0.27	0.10	0.044	0.083
DAG	0.58	0.51	0.50	0.55	0.43	0.098	0.838
MAG	0.14 ^b^	0.22 ^ab^	0.25 ^ab^	0.29 ^a^	0.18 ^ab^	0.030	0.013
FFA	5.08 ^a^	3.97 ^ab^	3.67 ^ab^	3.18 ^b^	3.10 ^b^	0.403	0.007
Upper Ileum							
TAG	0.09 ^b^	0.18 ^a^	0.19 ^a^	0.21 ^a^	0.14 ^ab^	0.017	<0.001
DAG	0.17 ^ab^	0.14 ^b^	0.20 ^ab^	0.27 ^a^	0.16 ^b^	0.024	0.007
MAG	0.11 ^c^	0.14 ^bc^	0.18 ^ab^	0.21 ^a^	0.11 ^c^	0.015	<0.001
FFA	2.67 ^a^	1.83 ^b^	1.61 ^bc^	1.71 ^bc^	1.20 ^c^	0.128	<0.001
Lower Ileum							
TAG	0.07 ^c^	0.11 ^bc^	0.15 ^ab^	0.18 ^a^	0.09 ^bc^	0.019	0.001
DAG	0.17	0.16	0.17	0.22	0.14	0.033	0.589
MAG	0.15 ^ab^	0.17 ^ab^	0.18 ^ab^	0.24 ^a^	0.13 ^b^	0.023	0.029
FFA	2.92 ^a^	1.62 ^b^	1.33 ^b^	1.40 ^b^	0.87 ^b^	0.241	<0.001
Excreta							
TAG	0.15 ^bc^	0.17 ^bc^	0.19 ^ab^	0.23 ^a^	0.12 ^c^	0.015	<0.001
DAG	0.15	0.13	0.17	0.22	0.13	0.026	0.111
MAG	0.13 ^cd^	0.15 ^bc^	0.19 ^ab^	0.21 ^a^	0.09 ^d^	0.014	<0.001
FFA	2.66 ^a^	1.50 ^b^	1.59 ^b^	1.66 ^b^	0.96 ^c^	0.119	<0.001

Abbreviations: TAG, triacylglycerols; DAG, diacylglycerols; MAG, monoacylglycerols; FFA, free fatty acids. ^1^ Lipid class concentration (mg/g)/Ti concentration (mg/g) in each intestinal segment and excreta. ^2^ Values are pooled means of 6 replicates with 2 chickens/replicate fed diets supplemented with 6% of palm oil (P), soybean acid oil (SA), soybean oil (S), or oil blends with 4% palm oil + 2% soybean acid oil (P4-SA2) or 2% palm oil + 4% soybean acid oil (P2-SA4). ^3^ SEM = standard error of the mean. ^a–d^: means in a row not sharing a common letter are significantly different (*p* < 0.05).

**Table 7:** Apparent metabolizable energy values and apparent fatty-acid digestibility coefficients in the different intestinal segments and total tract digestibility according to various fat sources in the diet in 11-d-old broiler chickens.

Item	Dietary Treatments ^1^					SEM ^4^	*p*-Value
	P6	P4-SA2	P2-SA4	SA6	S6		
AME, kcal/kg ^2^	3014 ^bc^	3109 ^bc^	3001 ^c^	3119 ^b^	3348 ^a^	27.783	<0.001
Upper Jejunum ^3^							
TFA	0.20 ^b^	0.33 ^ab^	0.12 ^b^	0.39 ^ab^	0.61 ^a^	0.081	<0.001
SFA	0.19	0.22	−0.02	−0.19	0.20	0.111	0.050
MUFA	0.31 ^ab^	0.36 ^ab^	0.22 ^ab^	0.20 ^b^	0.51 ^a^	0.078	0.028
PUFA	0.04 ^d^	0.41 ^bc^	0.15 ^cd^	0.57 ^ab^	0.78 ^a^	0.092	<0.001
Lower Jejunum ^3^							
TFA	0.48 ^c^	0.54 ^bc^	0.62 ^b^	0.71 ^a^	0.72 ^a^	0.022	<0.001
SFA	0.37 ^b^	0.37 ^b^	0.40 ^b^	0.46 ^ab^	0.60 ^a^	0.034	<0.001
MUFA	0.58 ^b^	0.58 ^b^	0.62 ^ab^	0.65 ^ab^	0.69 ^a^	0.026	0.017
PUFA	0.56 ^c^	0.69 ^b^	0.77 ^ab^	0.81 ^a^	0.77 ^ab^	0.024	<0.001
Upper Ileum ^3^							
TFA	0.51 ^c^	0.56 ^c^	0.62 ^bc^	0.72 ^ab^	0.74 ^a^	0.029	<0.001
SFA	0.36 ^b^	0.36 ^b^	0.40 ^b^	0.48 ^b^	0.65 ^a^	0.037	<0.001
MUFA	0.62	0.61	0.62	0.67	0.73	0.033	0.112
PUFA	0.63 ^b^	0.72 ^ab^	0.74 ^ab^	0.82 ^a^	0.78 ^a^	0.027	0.001
Lower Ileum ^3^							
TFA	0.62 ^c^	0.65 ^bc^	0.72 ^ab^	0.80 ^a^	0.79 ^a^	0.025	<0.001
SFA	0.49 ^b^	0.46 ^b^	0.51 ^b^	0.55 ^b^	0.69 ^a^	0.037	<0.001
MUFA	0.74	0.70	0.73	0.75	0.76	0.028	0.552
PUFA	0.75 ^c^	0.81 ^bc^	0.85 ^ab^	0.90 ^a^	0.83 ^abc^	0.023	0.001
Excreta ^3^							
TFA	0.60 ^c^	0.63 ^bc^	0.72 ^ab^	0.78 ^a^	0.80 ^a^	0.024	<0.001
SFA	0.39 ^b^	0.40 ^b^	0.43 ^b^	0.47 ^b^	0.64 ^a^	0.035	<0.001
MUFA	0.73	0.70	0.72	0.74	0.79	0.022	0.093
PUFA	0.80	0.80	0.83	0.91	0.85	0.029	0.094

Abbreviations: AME, apparent metabolizable energy; TFA, total fatty acids; SFA, saturated fatty acids; MUFA, monounsaturated fatty acids; PUFA, polyunsaturated fatty acids. ^1^ Diets supplemented with 6% of palm oil (P), soybean acid oil (SA), soybean oil (S), or oil blends with 4% palm oil + 2% soybean acid oil (P4-SA2) or 2% palm oil + 4% soybean acid oil (P2-SA4). ^2^ Values are pooled means of 6 replicates with 16 chickens/replicate until 11 d and 4 chickens/replicate until 22 d. ^3^ Values are pooled means of 6 replicates with 12 chickens/replicate. ^4^ SEM = standard error of the mean. ^a–d^: means in a row not sharing a common letter are significantly different (*p* < 0.05).

**Table 8:** Apparent metabolizable energy values and apparent fatty-acid digestibility coefficients in the different intestinal segments and total tract digestibility according to various fat sources in the diet in 35-d-old broiler chickens.

Item	Dietary Treatments ^1^					SEM ^4^	*p*-Value
	P6	P4-SA2	P2-SA4	SA6	S6		
AME, kcal/kg ^2^	3279 ^b^	3324 ^ab^	3384 ^a^	3274 ^b^	3364 ^ab^	23.350	0.008
Upper Jejunum ^3^							
TFA	0.51	0.44	0.45	0.35	0.48	0.052	0.283
SFA	0.48 ^a^	0.40 ^a^	0.36 ^ab^	0.11 ^b^	0.21 ^ab^	0.073	0.005
MUFA	0.67	0.60	0.59	0.49	0.60	0.043	0.059
PUFA	0.34	0.35	0.42	0.38	0.51	0.060	0.285
Lower Jejunum ^3^							
TFA	0.69 ^b^	0.74 ^ab^	0.73 ^ab^	0.72 ^ab^	0.81 ^a^	0.024	0.019
SFA	0.64	0.71	0.72	0.62	0.73	0.032	0.061
MUFA	0.83 ^a^	0.83 ^a^	0.79 ^ab^	0.74 ^b^	0.85 ^a^	0.019	0.002
PUFA	0.56 ^c^	0.69 ^b^	0.70 ^b^	0.74 ^ab^	0.81 ^a^	0.025	<0.001
Upper Ileum ^3^							
TFA	0.82 ^c^	0.84 ^bc^	0.88 ^a^	0.86 ^ab^	0.89 ^a^	0.009	<0.001
SFA	0.77 ^b^	0.81 ^ab^	0.85 ^a^	0.77 ^b^	0.85 ^a^	0.015	<0.001
MUFA	0.91 ^a^	0.87 ^a^	0.90 ^a^	0.86 ^b^	0.91 ^a^	0.007	<0.001
PUFA	0.78 ^c^	0.82 ^b^	0.89 ^a^	0.89 ^a^	0.90 ^a^	0.008	<0.001
Lower Ileum ^3^							
TFA	0.84 ^b^	0.88 ^ab^	0.91 ^a^	0.91 ^a^	0.92 ^a^	0.010	<0.001
SFA	0.78 ^b^	0.85 ^a^	0.89 ^a^	0.85 ^a^	0.90 ^a^	0.016	<0.001
MUFA	0.93	0.92	0.91	0.90	0.93	0.011	0.282
PUFA	0.83 ^c^	0.88 ^b^	0.92 ^ab^	0.93 ^a^	0.93 ^a^	0.011	<0.001
Excreta ^3^							
TFA	0.84 ^c^	0.90 ^b^	0.90 ^ab^	0.89 ^b^	0.93 ^a^	0.006	<0.001
SFA	0.77 ^b^	0.86 ^a^	0.85 ^a^	0.79 ^b^	0.87 ^a^	0.009	<0.001
MUFA	0.92 ^ab^	0.92 ^a^	0.91 ^b^	0.87 ^c^	0.93 ^a^	0.004	<0.001
PUFA	0.84 ^c^	0.91 ^b^	0.93 ^ab^	0.94 ^ab^	0.94 ^a^	0.007	<0.001

Abbreviations: AME, apparent metabolizable energy; TFA, total fatty acids; SFA, saturated fatty acids; MUFA, monounsaturated fatty acids; PUFA, polyunsaturated fatty acids. ^1^ Diets supplemented with 6% of palm oil (P), soybean acid oil (SA), soybean oil (S), or oil blends with 4% palm oil + 2% soybean acid oil (P4-SA2) or 2% palm oil + 4% soybean acid oil (P2-SA4). ^2^ Values are pooled means of 6 replicates with 4 chickens/replicate. ^3^ Values are pooled means of 6 replicates with 2 chickens/replicate. ^4^ SEM = standard error of the mean. ^a–c^: means in a row not sharing a common letter are significantly different (*p* < 0.05).

## Supplementary Materials

The following are available online at https://www.mdpi.com/article/10.3390/ani11092586/s1. Table S1. Lipid-class content according to different intestinal segments and excreta in 11- and 35-day-old broiler chickens. Table S2. Lipid-class content in the lower ileum according to different fat sources in the diet in 11- and 35-day-old broiler chickens. Table S3. Feed apparent metabolizable energy value and apparent fatty-acid digestibility coefficients in the lower ileum according to different fat sources in the diet in 11- and 35-day-old broiler chickens. Table S4. Contribution of each intestinal segment to FA absorption according to the age of the chicken.
