# Supplementary material for: Replacement of Palm Oil with Soybean Acid Oil in Broiler Chicken Diet: Fat Digestibility and Lipid Class Content along the Intestinal Tract
Source: Animals (Basel). 2021 Sep 3;11(9):2586. doi: 10.3390/ani11092586 (PMC8469231; doi:10.3390/ani11092586)
Supplement: Supplementary file 1 [file animals-11-02586-s001.zip › animals-1306033-supplementary.pdf]

animals

MDPI

Table S1. Lipid-class content[1] according to different intestinal segments and excreta in 11- and 35-day-old broiler chickens.

	Intestinal Segment and Excreta [2]					p-Values	
**Item**	**Upper Jejunum**	**Lower Jejunum**	**Upper Ileum**	**Lower Ileum**	**Excreta**	**SEM**	**P**
**11-day-old broiler chickens**							
TAG	0.57 [a]	0.41 [b]	0.29 [b]	0.31 [b]	0.38 [b]	0.039	<0.001
DAG	2.12 [a]	0.89 [b]	0.65 [b]	0.61 [b]	0.96 [b]	0.103	<0.001
MAG	0.25	0.30	0.26	0.34	0.30	0.025	0.104
FFA	13.46 [a]	6.23 [b]	5.40 [b]	5.22 [b]	6.82 [b]	0.677	<0.001
**35-day-old broiler chickens**							
TAG	0.24 [a]	0.22 [ab]	0.16 [bc]	0.11 [c]	0.17 [bc]	0.016	<0.001
DAG	1.47 [a]	0.52 [b]	0.19 [c]	0.17 [c]	0.16 [c]	0.042	<0.001
MAG	0.27 [a]	0.22 [ab]	0.15 [c]	0.18 [bc]	0.15 [c]	0.016	<0.001
FFA	7.96 [a]	3.82 [b]	1.80 [c]	1.63 [c]	1.67 [c]	0.210	<0.001

[1] Lipid-class concentration (mg/g)/Ti concentration (mg/g). [2]Values are pooled means of 30 replicates from chickens fed diets at 11-day-old (n = 150) or 35-day-old (n = 150). TAG = triacylglycerols; DAG = diacylglycerols; MAG = monoacylglycerols; FFA = free fatty acids. SEM = standard error of the mean. p-Values were obtained from univariate ANOVA conducted for each age to study whether the intestinal segment (or excreta) affected the lipid-class content. a-c: means in a row not sharing a common letter are significantly different ($p < 0.05$).

Table S2. Lipid-class content in the lower ileum according to different fat sources in the diet in 11- and 35-day-old broiler chickens.

	Dietary Treatments [1]																			
	11-day-old broiler chickens					35-day-old broiler chickens														
**Item**	**P6**	**P4-SA2**	**P2-SA4**	**SA6**	**S6**	**P6**	**P4-SA2**	**P2-SA4**	**SA6**	**S6**	**P6**		**P4-SA2**		**P2-SA4**		**SA6**		**S6**	
											**SEM**	**P**	**SEM**	**P**	**SEM**	**P**	**SEM**	**P**	**SEM**	**P**
TAG	0.19	0.30	0.36	0.38	0.32	0.07	0.11	0.15	0.18	0.09	0.021	0.002	0.019	<0.001	0.037	0.002	0.052	0.012	0.020	<0.001
DAG	0.45	0.58	0.72	0.68	0.64	0.17	0.16	0.17	0.22	0.14	0.071	0.021	0.045	<0.001	0.058	<0.001	0.070	<0.001	0.069	<0.001
MAG	0.15	0.35	0.45	0.52	0.23	0.15	0.17	0.18	0.24	0.13	0.019	1.000	0.023	<0.001	0.027	<0.001	0.047	0.002	0.014	<0.001
FFA	8.47	6.83	4.63	3.17	3.02	2.92	1.62	1.33	1.40	0.87	0.574	<0.001	0.298	<0.001	0.278	<0.001	0.208	<0.001	0.252	<0.001

[1]Values are pooled means of 6 replicates from chickens fed diets supplemented with 6% of palm oil (P), soybean acid oil (SA), soybean oil (S), or oil blends with 4% palm oil and 2% soybean acid oil (P4-SA2) or 2% palm oil and 4% soybean acid oil (P2-SA4). TAG = triacylglycerols; DAG = diacylglycerols; MAG = monoacylglycerols; FFA = free fatty acids. SEM = standard error of the mean. P = *p*-values, that were obtained from univariate ANOVA conducted for each dietary treatment to study whether the age affected the lipid-class content ($n = 12$). $p < 0.05$ was considered significant.

Table S3. Feed apparent metabolizable energy value and apparent fatty-acid digestibility coefficients in the lower ileum according to different fat sources in the diet in 11- and 35-day-old broiler chickens.

	Dietary Treatments [1]																			
	11-day-old broiler chickens					35-day-old broiler chickens														
											P6		P4-SA2		P2-SA4		SA6		S6	
**Item**	**P6**	**P4-SA2**	**P2-SA4**	**SA6**	**S6**	**P6**	**P4-SA2**	**P2-SA4**	**SA6**	**S6**	**SEM**	**P**	**SEM**	**P**	**SEM**	**P**	**SEM**	**P**	**SEM**	**P**
AME, kcal/kg[2]	3014	3109	3001	3119	3348	3279	3324	3384	3274	3364	31.27	<0.001	26.57	<0.001	20.32	<0.001	24.26	0.001	24.64	0.656
FA digestibility																				
TFA	0.62	0.65	0.72	0.80	0.79	0.84	0.88	0.91	0.91	0.92	0.022	<0.001	0.016	<0.001	0.019	<0.001	0.017	<0.001	0.011	<0.001
SFA	0.49	0.46	0.51	0.55	0.69	0.78	0.85	0.89	0.85	0.90	0.026	<0.001	0.021	<0.001	0.034	<0.001	0.028	<0.001	0.011	<0.001
MUFA	0.74	0.70	0.73	0.75	0.76	0.93	0.92	0.91	0.90	0.93	0.026	<0.001	0.017	<0.001	0.024	<0.001	0.022	<0.001	0.010	<0.001
PUFA	0.75	0.81	0.85	0.90	0.83	0.83	0.88	0.92	0.93	0.93	0.028	0.056	0.013	0.002	0.017	0.014	0.016	0.234	0.011	<0.001

[1]Values are pooled means of 6 replicates from chickens fed diets supplemented with 6% of palm oil (P), soybean acid oil (SA), soybean oil (S), or oil blends with 4% palm oil and 2% soybean acid oil (P4-SA2) or 2% palm oil and 4% soybean acid oil (P2-SA4). AME = apparent metabolizable energy, TFA = total fatty acids, SFA = saturated fatty acids, MUFA = monounsaturated fatty acids, PUFA = polyunsaturated fatty acids, SEM = standard error of the mean. P = *p*-values, that were obtained from univariate ANOVA conducted for each dietary treatment to study whether the age affected the feed AME values, and the FA digestibility results (n = 12). $p < 0.05$ was considered significant.

Table S4. Contribution of each intestinal segment to FA absorption according to the age of the chicken.

	Age (days) [1]			
**Item**	**11**	**35**	**SEM**	***p*–Value**
**Upper jejunum**				
TFA	54.96	49.83	4.198	0.347
Palmitic	34.24	49.36	4.736	0.019
Stearic	-	-	-	-
Oleic	47.89	64.45	3.731	0.002
Linoleic	54.93	44.55	4.999	0.124
**Lower jejunum**				
TFA	42.50	33.04	4.347	0.117
Palmitic	51.40	34.38	4.286	0.005
Stearic	58.31	59.05	4.160	0.896
Oleic	40.16	24.26	3.448	0.001
Linoleic	41.80	33.51	4.697	0.206
**Upper ileum**				
TFA	7.03	13.01	2.296	0.064
Palmitic	8.67	11.92	2.277	0.300
Stearic	16.17	27.34	2.880	0.007
Oleic	9.01	8.40	2.248	0.844
Linoleic	6.08	17.09	1.575	<0.001
**Lower ileum**				
TFA	10.02	4.11	0.720	<0.001
Palmitic	10.76	4.34	1.045	<0.001
Stearic	26.25	9.93	2.859	<0.001
Oleic	9.17	2.89	0.871	<0.001
Linoleic	8.39	4.84	0.790	0.002

[1]Values are means of 30 replicates from chickens fed diets at 11-day-old or 35-day-old. TFA = total fatty acids. SEM = standard error of the mean. p-Values were obtained from univariate ANOVA conducted for each intestinal segment to study whether the age affected the FA absorption (n = 60). $p < 0.05$ was considered significant.
